# Supplementary material for: Dual-Activated Nano-Prodrug for Chemo-Photodynamic Combination Therapy of Breast Cancer
Source: Int J Mol Sci. 2022 Dec 10;23(24):15656. doi: 10.3390/ijms232415656 (PMC9779597; doi:10.3390/ijms232415656)
Supplement: Supplementary file 1 [file ijms-23-15656-s001.zip › ijms-2011870-supplementary.pdf]

Supporting information

Dual-activated nanoprodrug for chemo-photodynamic combination therapy of breast cancer

Zi-Yao Lu [a, b, ‡] Gan Xu [c, ‡], Xiao-Zhen Yang [c], Shi-Jia Liu [a], Yang Sun [a, d], Li Chen [a, b], Qin-Ying Liu [a, *], Jian-Yong Liu [c, e, f, *]

*Corresponding authors. E-mail: liuqinyingbio@163.com (Q. Liu), lkw82@fzu.edu.cn (J.-Y. Liu).

‡These authors contributed equally to this work.

Table of Content

Materials and instruments

Determination of BTC and BCC Loading Content

Measurement of fluorescence quantum yield

Singlet oxygen generation efficiency investigation

Figure S1. Electronic absorption spectra of CPT, **2**, BCC, and BTC in DMSO.

Figure S2. Electronic absorption spectra of BTC and BCC in DMSO.

Table S1. Optical data for the BODIPY derivatives

Figure S3. Effects of GSH on fluorescence emission of BTC and BCC in a mixture of DMSO and PBS (v/v, 3:1).

Figure S4. Effects of GSH on ROS production of BTC and BCC in a mixture of DMSO and PBS (v/v, 3:1).

Figure S5. Fluorescence emission spectra of CPT, **2**, BCC, and BTC in DMSO.

Figure S6. Light-induced CPT release from BTC and BCC in DMSO/PBS (v/v, 3:1) under different conditions.

Figure S7. Preparation and Characterization of BCC NPs.

Figure S8. Intracellular ROS production and GSH-responsive properties of BTC NPs and BCC NPs in MCF-7 cells.

Figure S9. The intracellular CPT release and cytotoxicity from BTC NPs and BCC NPs in MCF-7 cells under light irradiation.

Figure S10. *In vivo* fluorescence images of 4T1-bearing mice at different time-points after intravenous injection of BTC and BCC.

Figure S11. H&E-stained images of main organs from 4T1 tumor-bearing mice after treatment with BTC, BCC, or saline under light illumination.

Figure S12. ^{1}H NMR spectrum of BTC.

Figure S13. ^{13}C NMR spectrum of BTC.

Figure S14. HRMS spectrum of BTC.

Figure S15. ^{1}H NMR spectrum of BCC.

Figure S16. ^{13}C NMR spectrum of BCC.

Figure S17. HRMS spectrum of BCC.

Materials and instruments

All solvents were obtained from Titan Scientific Co., Ltd., Shanghai, China. MCF-7 human breast cancer and 4T1 mouse mammary carcinoma cells were obtained from the cell bank of Shanghai Institutes for Biological Sciences. Dulbecco's Modified Eagle Medium (DMEM), fetal bovine serum (FBS), penicillin-streptomycin, and trypsin were purchased from Thermo Fisher Scientific (China) Co., Ltd., Shanghai, China. Confocal dish and 96-well sterile cell culture plate were purchased from NEST Biotechnology Co., Ltd., Wuxi, Jiangsu, China. 2',7'-dichlorofluorescein diacetate (DCFH-DA), 3-(4,5-dimethylthiazol-2-yl)-2,5-diphenyltetrazolium bromide (MTT), and L-buthionine sulfoximine (BSO) were obtained from Dingguo Biotechnology Co., Ltd., Haimen, China.

^{1}H NMR spectra were conducted on a AVANCE Ⅲ (^{1}H, 400 MHz) instrument (Bruker, Karlstuhe, Germany) in $CDCl_3$. Nanoparticle size was measured on a Zetasizer NanoZS dynamic light scattering instrument (Malvern Instruments, Worcestershire, U.K.) and transmission electron microscope (JEM-2100F, JEOL, Japan). Electronic absorption and Fluorescence emission spectra were performed on the PerkinElmer Lambda 365 UV-Visible absorption spectrometer (Massachusetts, USA) and VARIAN Carye Elipse Fluorescence Spectrometer (California, USA), respectively. Intracellular fluorescence imaging was carried out on Olympus FV1000 confocal laser scanning microscope. The intracellular were carried on Olympus FV1000 confocal laser scanning microscope (Tokyo, Japan). In vivo fluorescence imaging was recorded on FMTTM 2500LX Fluorescence Molecular Tomography (PerkinElmer, Waltham, MA).

MCF-7 and 4T1 cells were cultured in DMEM medium containing 10% FBS and 1% penicillin-streptomycin at 37°C in a humidified incubator with a 5% CO_2atmosphere. Female BALB/c mice (20 g-25 g) were purchased from the Wushi Laboratory Animal (Fuzhou, China). All animal experiments were approved by the Animal Ethics Committee of Fuzhou University.

Determination of BTC and BCC Loading Content

To measure the amount of BTC (BCC) in BTC NPs (BCC NPs), the NPs were

dissolved in dimethyl sulfoxide (DMSO) and the absorption values at 683 nm were recorded. The loaded amount of BTC or BCC was calculated from calibration curves by UV-Vis spectrophotometry. Loading Content (LC) (wt%) = (mass of loaded BTC or BCC/total mass of polymer and loaded drug) × 100%; Loading Efficiency (LE) (wt%) = (mass of loaded BTC or BCC/mass of BTC or BCC in feed) × 100%.

Measurement of fluorescence quantum yield

The PSs (1, 2, BTC, and BCC) were dissolved in DMSO to afford 1 mM stock solutions, and then their fluorescence emission spectra were measured when the absorbances of these samples at 610 nm were adjusted to the range of 0.04-0.05 by diluting the corresponding stock solutions. Fluorescence quantum yields (Φ_F) of these PSs in DMSO were calculated by the following formula:

$$\Phi_{F\ (sample)} = \Phi_{F\ (ref)} \times (A_{ref}/A_{sample}) \times (F_{sample}/F_{ref}) \times (\eta^2_{sample}/\eta^2_{ref})$$

Where A, F, and η are the absorbance at 610 nm, the measured fluorescence area under emission peak (λ_{ex} = 610 nm), and refractive index of the solution, respectively. Here, unsubstituted zinc(II) phthalocyanine (ZnPc) in DMSO was used as the reference ($\Phi_{F\ (ref)}$ = 0.20).

Singlet oxygen generation efficiency investigation

The $^1O_2$ generation efficiency of PSs was detected by using 1,3-diphenylisobenzofuran (DPBF) as the $^1O_2$ probe. Firstly, the samples (BTC and BCC) (2.5 mM) were incubated with GSH (0 mM, 2.5 μM, and 2.5 mM) in a mixture of DMSO and PBS (3:1, v/v) for 2 h. Then 15 mL of DPBF stock solution (5 mM) in DMSO were taken with a pipette gun and added into 3 mL of the above sample solutions, followed by laser irradiation (660 nm, 1 mW/cm^2). The electronic absorption spectra of these mixture in the range of 300-800 nm were recorded after each irradiation for 1 min, and repeated eleven times.

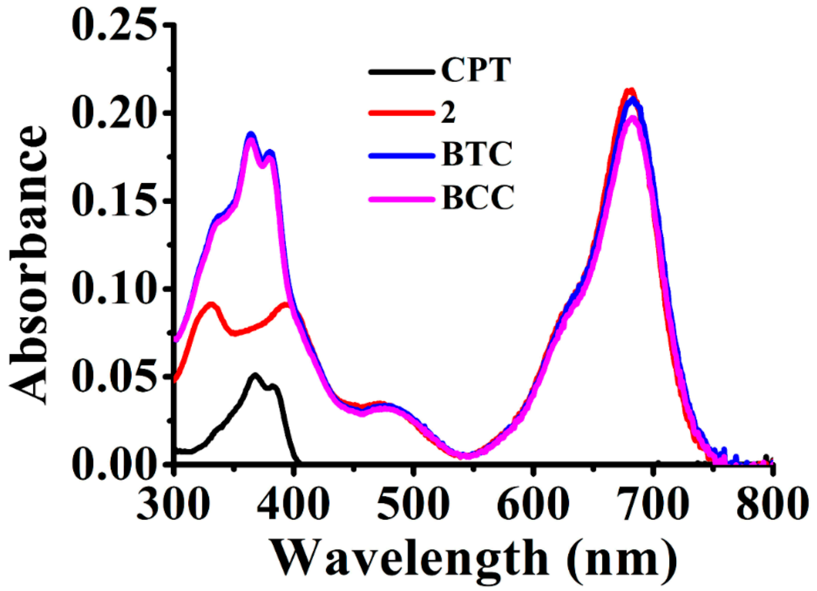

Figure S1. Electronic absorption spectra of BCC, BTC, **2**, and CPT (2 μM) in DMSO.

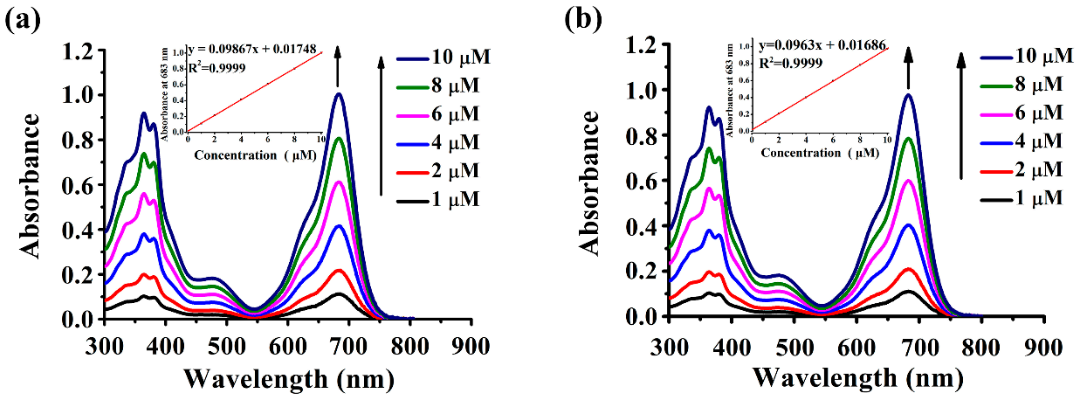

Figure S2. Electronic absorption spectra of (a) BTC and (b) BCC at different concentrations in DMSO. The inset plots the Q-band absorbance versus the concentration of compounds.

Table S1. Optical data of **1**, **2**, BCC, and BTC in DMSO

Compounds	λ_{max}^{abs}(nm)	ε(L•mol^{-1}•cm^{-1})	λ_{max}^{em}(nm)[a]	Φ_F[b]
1	673		718	0.319
2	683	-	724	0.081
BCC	683	98670	724	0.076
BTC	683	98670	724	0.078

[a] Excited at 610 nm.

[b] Using unsubstituted zinc(II) phthalocyanine (ZnPc) in DMSO as the reference (Φ_F = 0.20)

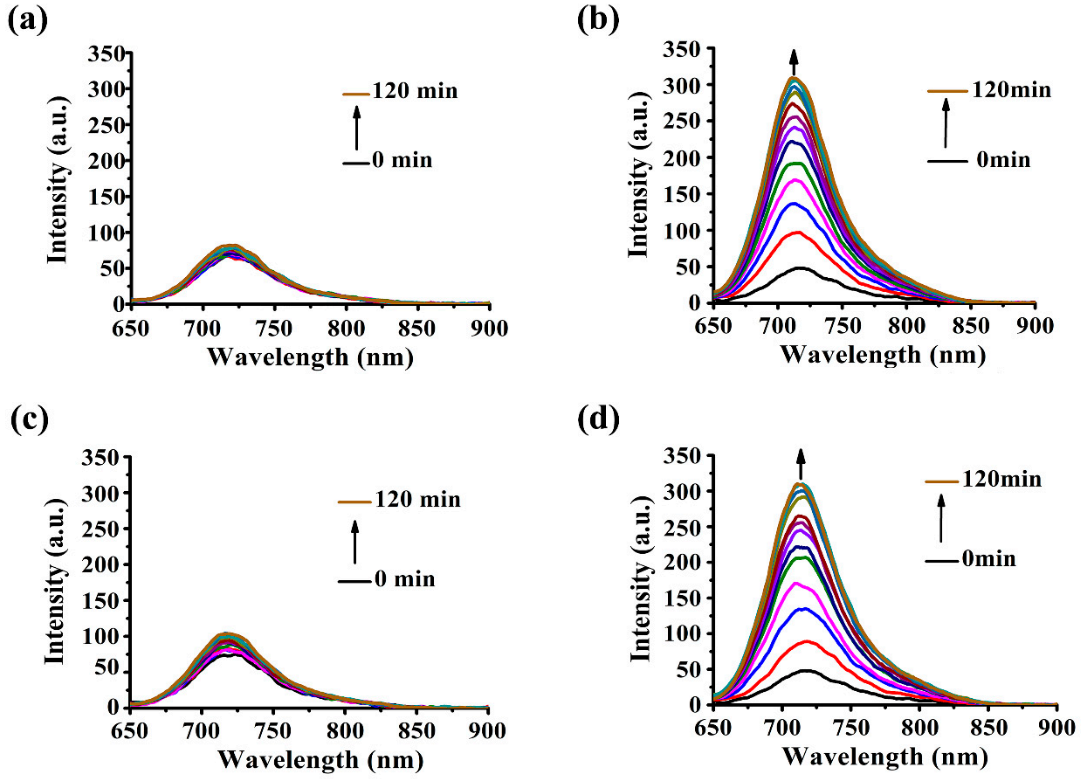

Figure S3. Changes of fluorescence emission spectra of BTC (a, b) and BCC (c, d) with incubation time upon addition of GSH (2.5 μM and 2.5 mM) in mixed solutions (DMSO: PBS =3:1), both BCC and BTC at 2.5 μM.

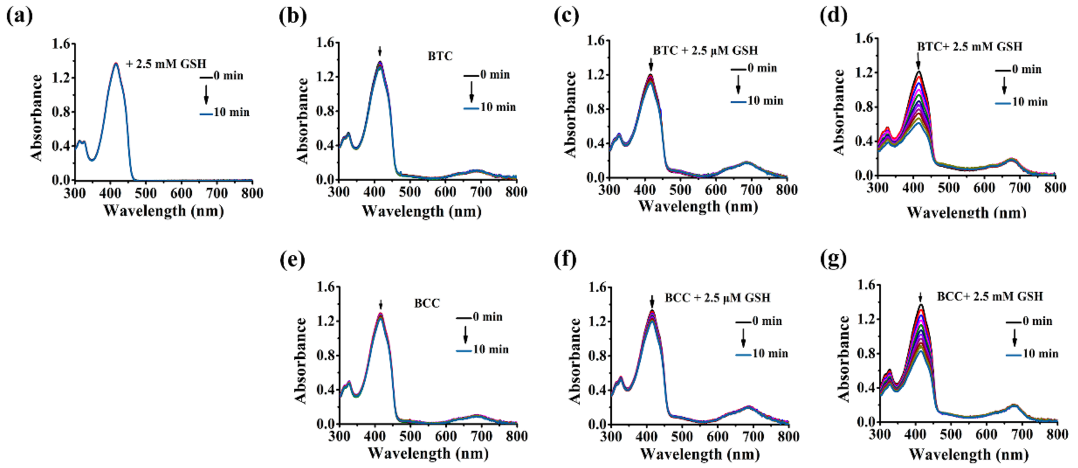

Figure S4. The electronic absorption spectra of a mixture of (a) GSH, (b) BTC, (c) BTC + 2.5 μM GSH, (d) BTC + 2.5 mM GSH, (e) BCC, (f) BCC + 2.5 μM GSH, (g) BTC + 2.5 mM GSH, and DPBF in mixed solutions (DMSO: PBS = 3:1) under laser irradiation (660 nm, 1 mW/cm^2) for 10 min (both BTC and BCC at 2.5 μM).

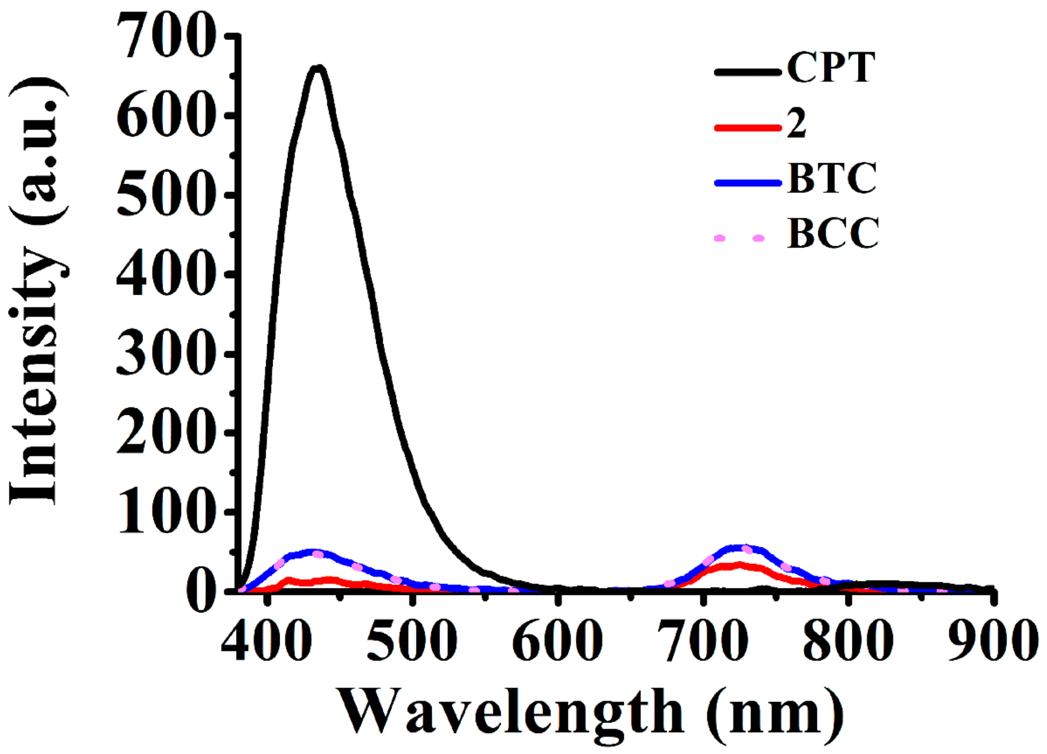

Figure S5. Fluorescence emission spectra of BCC, BTC, **2**, and CPT (2 μM) in DMSO.

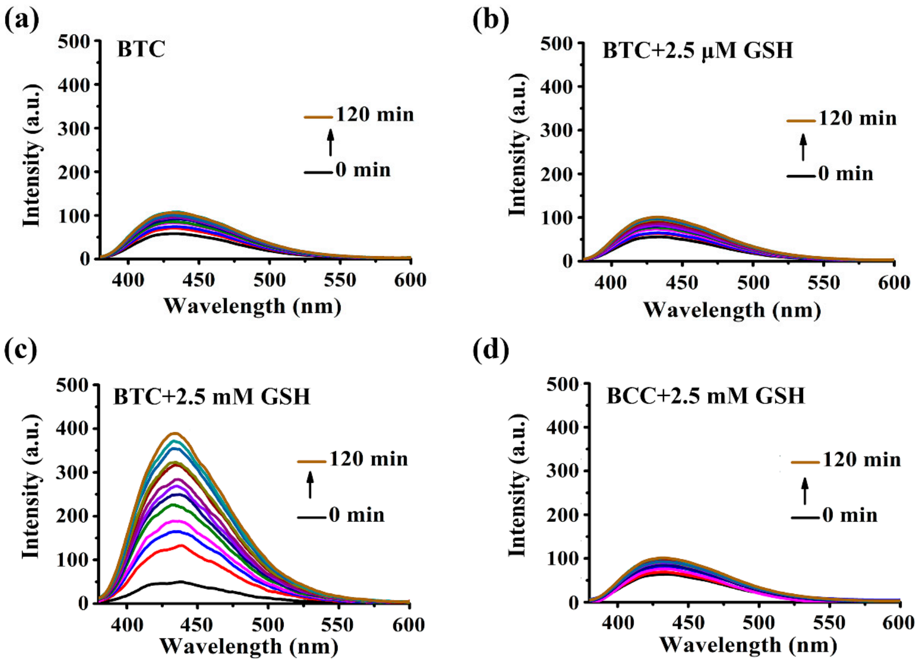

Figure S6. Changes of fluorescence spectra for (a) BTC, (b) BTC + 2.5 μM GSH, (c) BTC + 2.5 mM GSH, (d) BTC + 2.5 mM GSH in mixed solutions (DMSO: PBS = 3:1) after durations of laser irradiation (660 nm, 5 mW/cm^2).

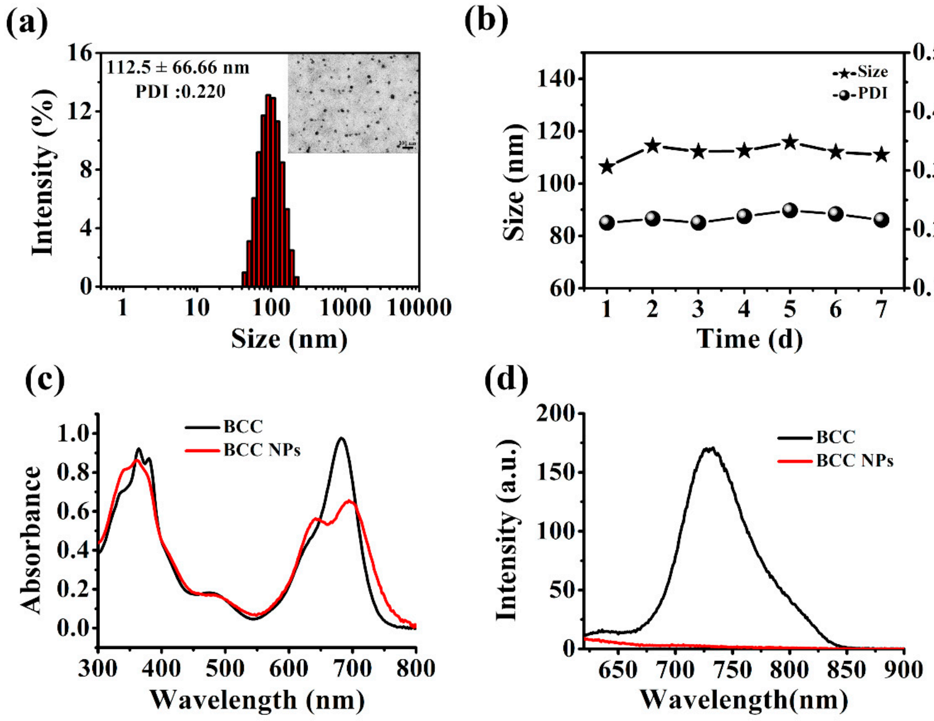

Figure S7. Preparation and characterization of BCC NPs. (a) Particle size distribution and TME image. (b) The change of particle size and PDI in PBS for 7 days. (c) UV–vis absorption spectra of BCC in DMSO and BCC NPs in PBS (both at 10 μM). (d) Fluorescence emission spectra of BCC in DMSO and BCC NPs in PBS (both at 10 μM).

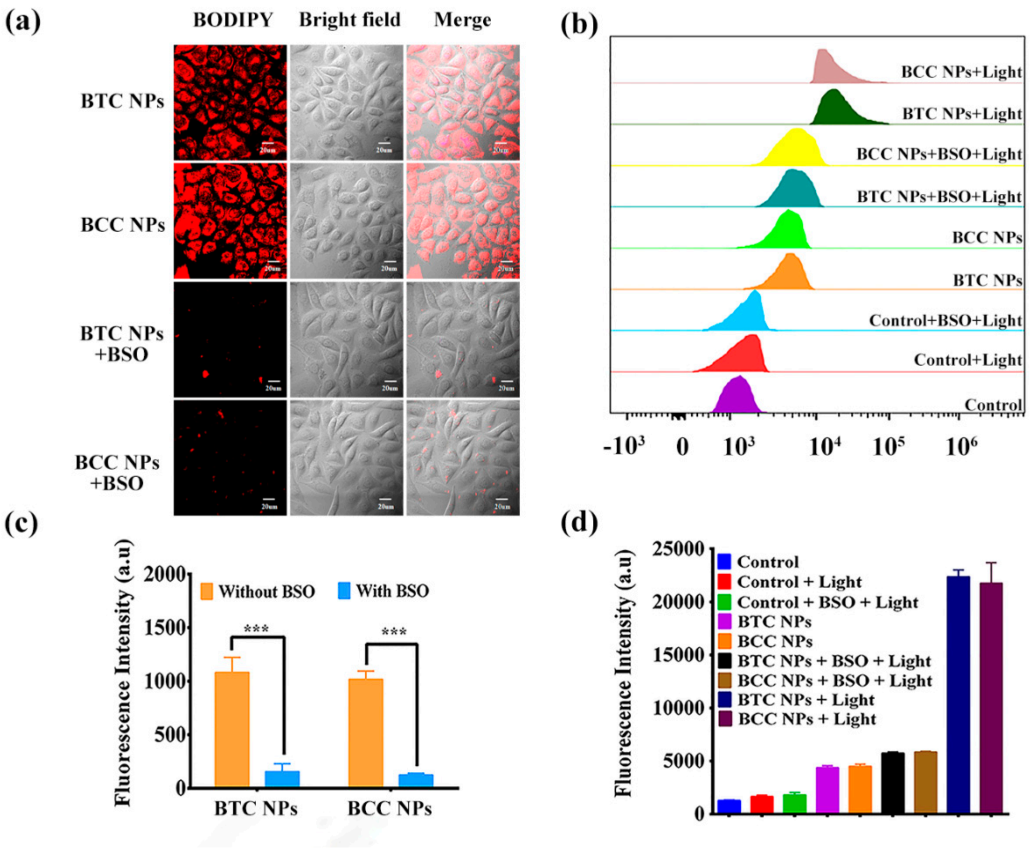

Figure S8. (a) Confocal laser scanning microscopy (CLSM) images of MCF-7 cells after incubation with BTC NPs (5 μM) for 24 h, or cells pre-treated with BSO (10 mM) for 12 h. (c) Quantitative results of (a) (*p < 0.05, **p < 0.01, ***p < 0.001). (b) ROS generation of 4T1 cells after incubation with laser irradiation at 660 nm (20 mW/cm^2, 5 min) by flow cytometry. (d) Quantitative results of (b).

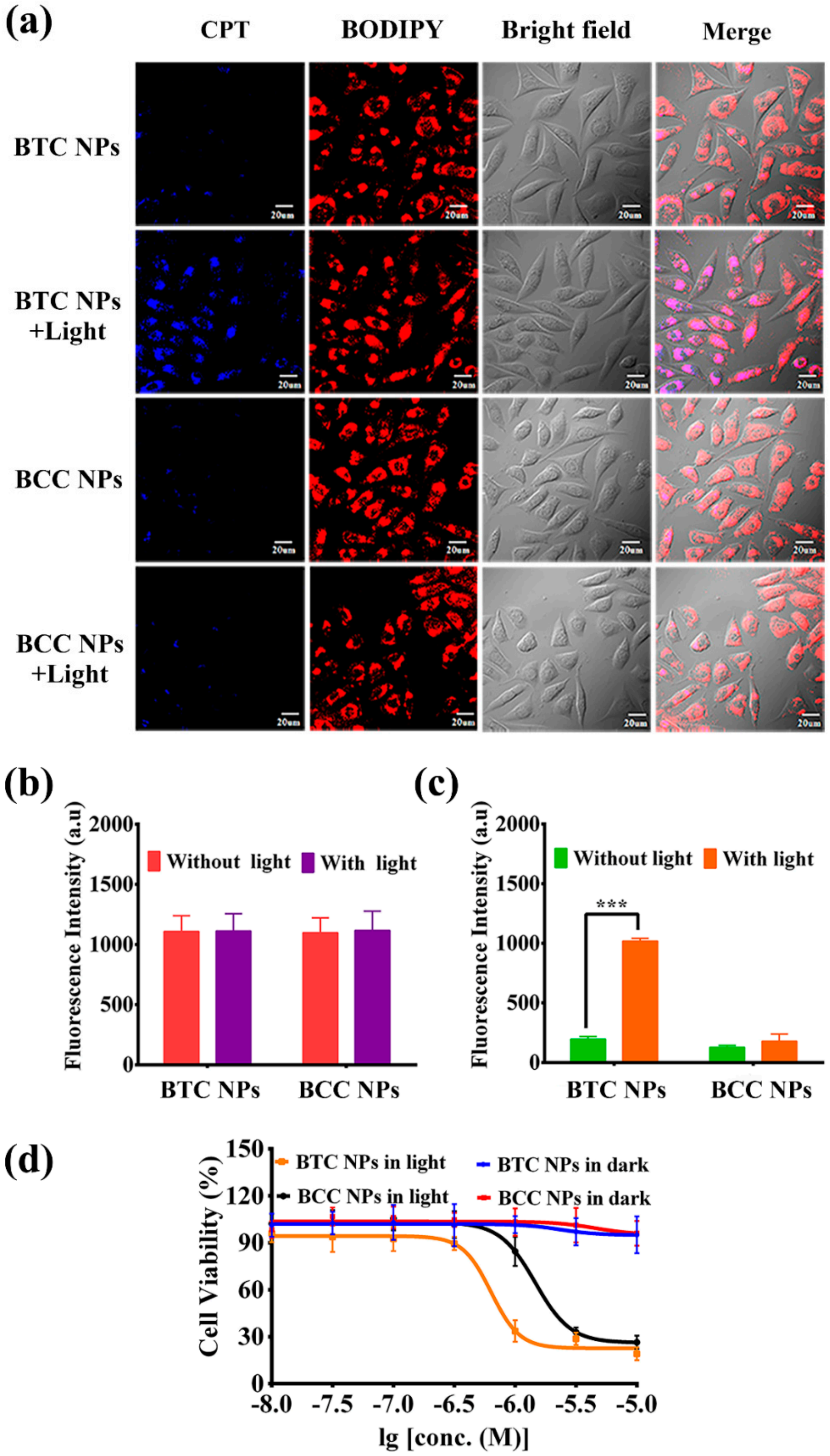

Figure S9. (a) Confocal laser scanning microscopy (CLSM) images of MCF-7 cells after incubation with BTC NPs (5 μM) for 24 h, or cells with 660 nm laser irradiation for incubation. (b) and (c) Quantitative results of (a) (*$p < 0.05$, **$p < 0.01$, ***$p < 0.001$). (d) *In vitro* cytotoxicity of BTC NPs against MCF-7 cells with or without laser irradiations (660 nm, 20 mW/cm^2, 5 min).

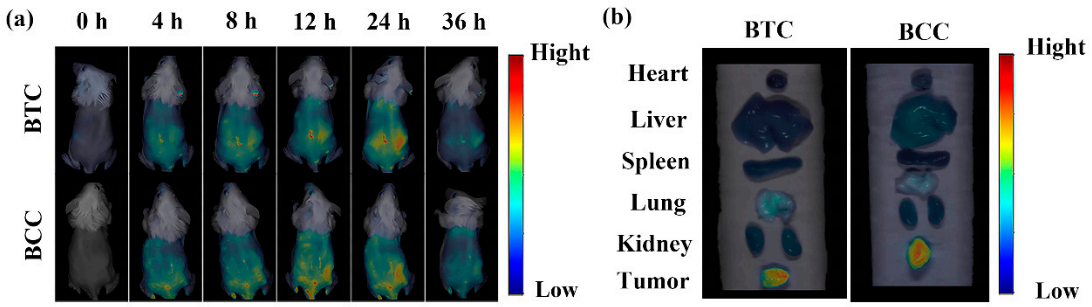

Figure S10. (a) Fluorescence images of 4T1 tumor-bearing mice at different time points after treatment with BTC NPs, BCC NPs, and saline. (b) The Ex vivo fluorescence images of the different organs and tumors at 12 h after intravenous injection with BTC (or BCC).

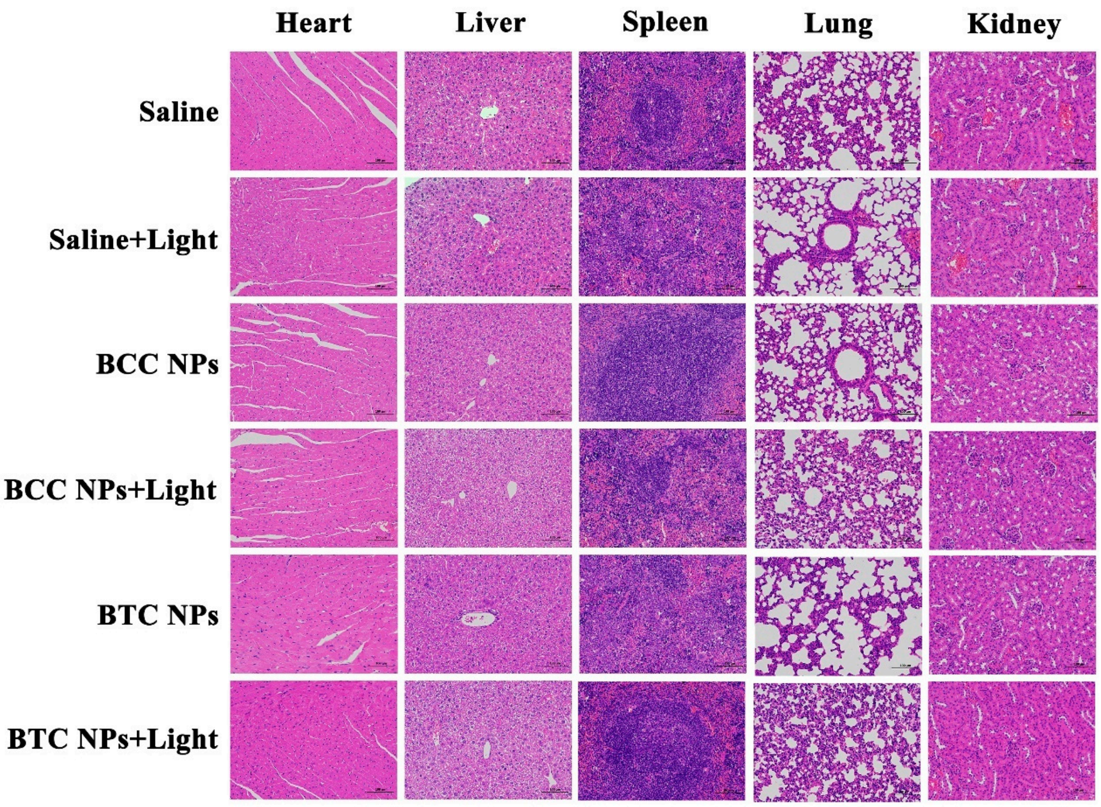

Figure S11. H&E-stained images of main organs from tumor-bearing mice after treatment with saline, BCC NPs, or BTC NPs with or without light irradiation. The scan bars were 50 μm.

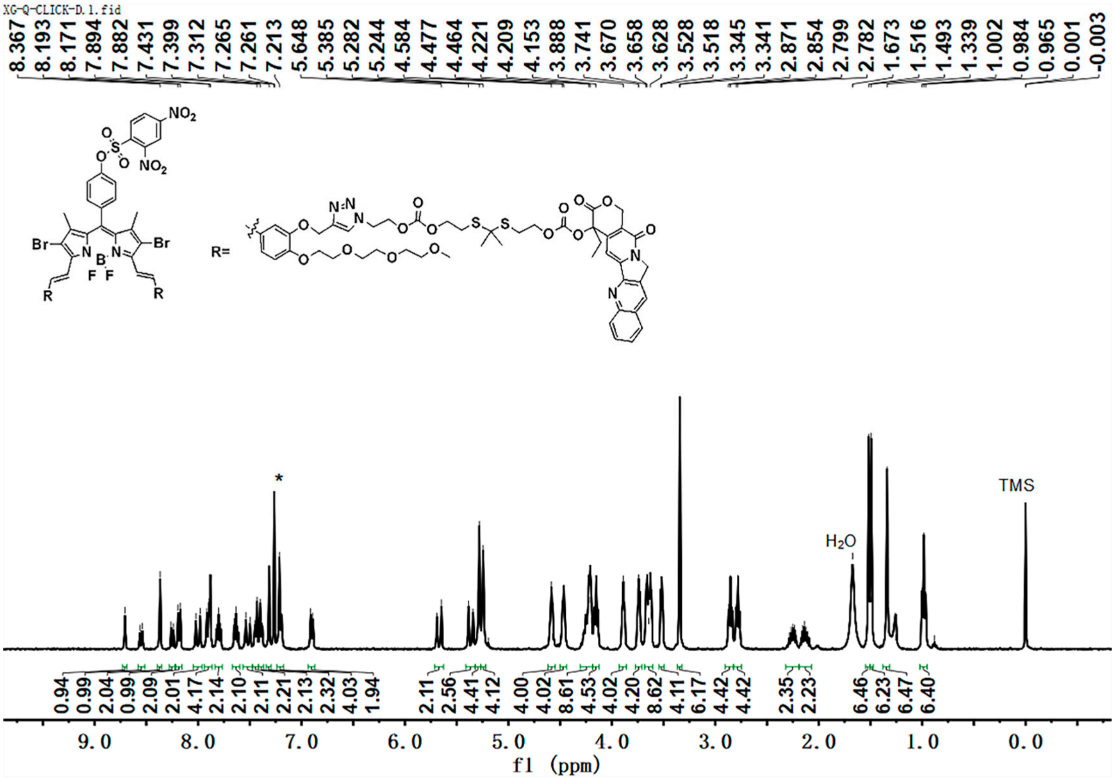

Figure. S12. ^{1}H NMR spectrum of BTC in $CDCl_3$.

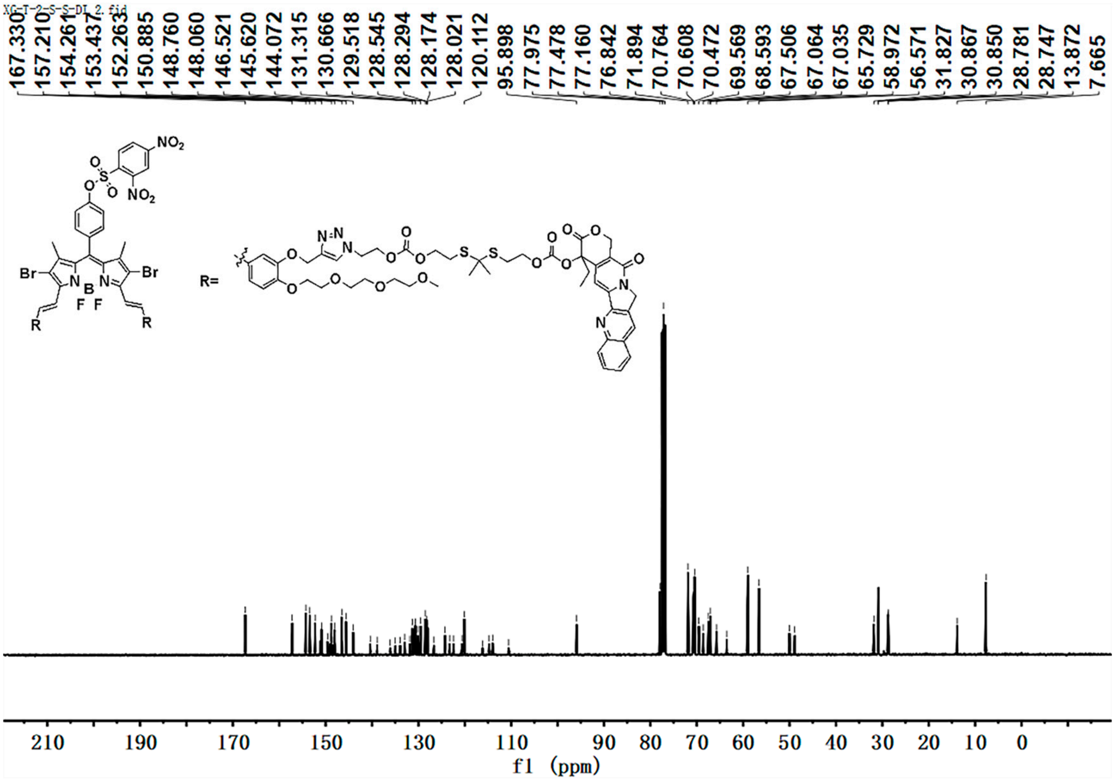

Figure S13. ^{13}C NMR spectrum of BTC in $CDCl_3$.

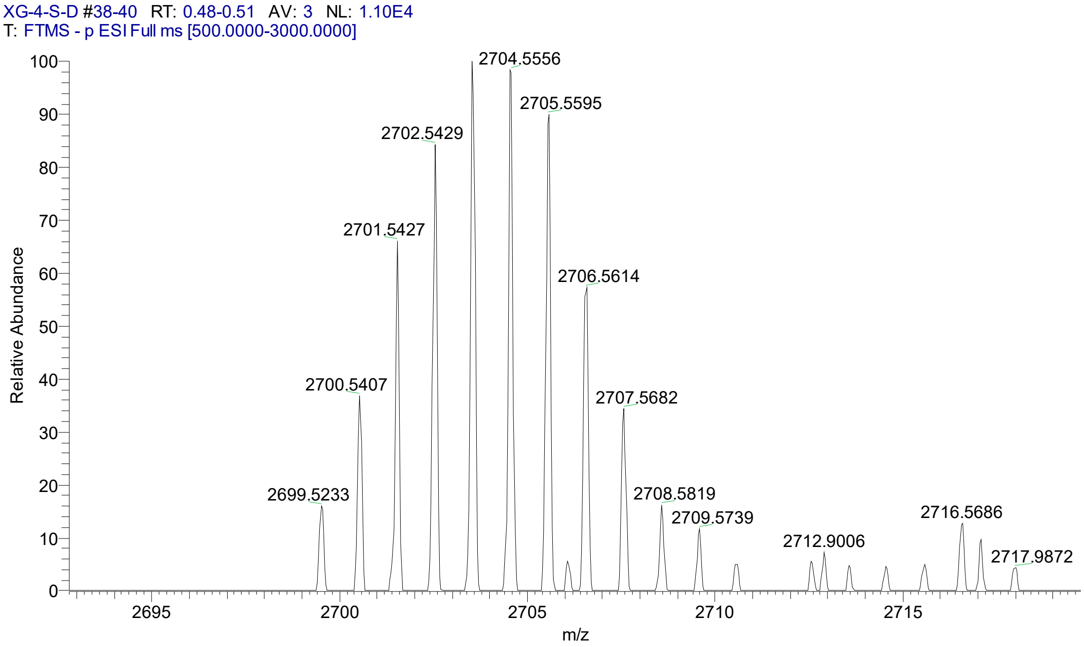

Figure. S14. HRMS spectrum of BTC.

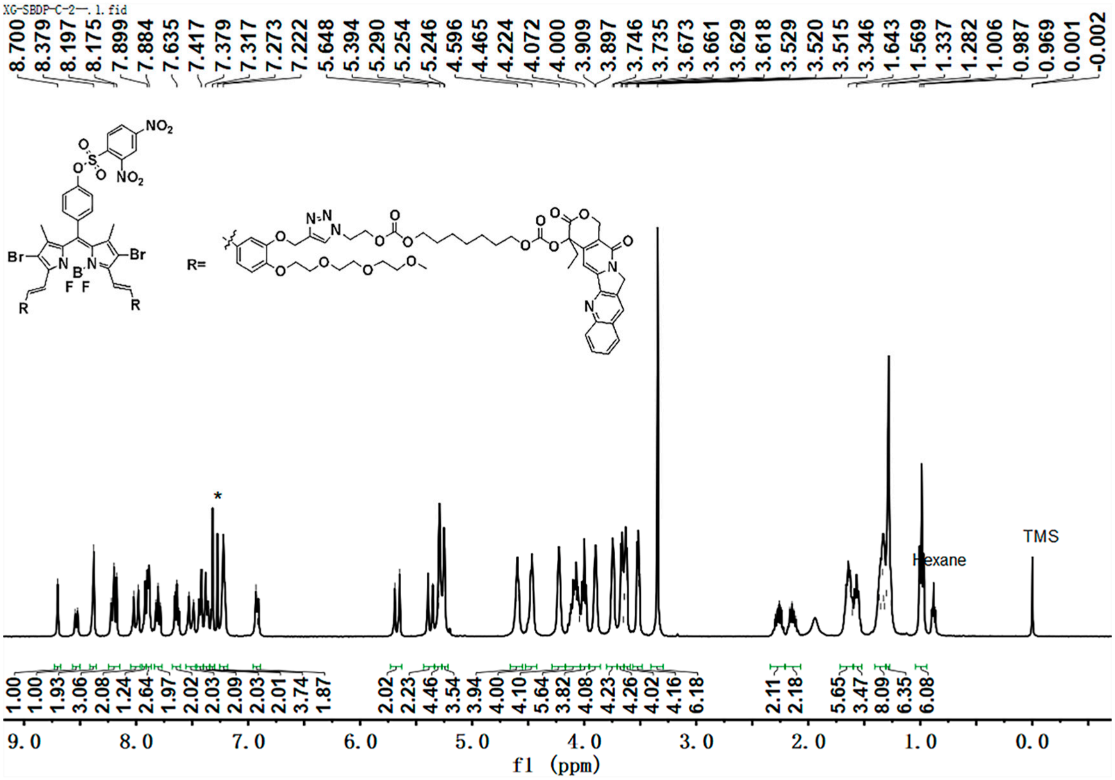

Figure S15. ^{1}H NMR spectrum of BCC in $CDCl_3$.

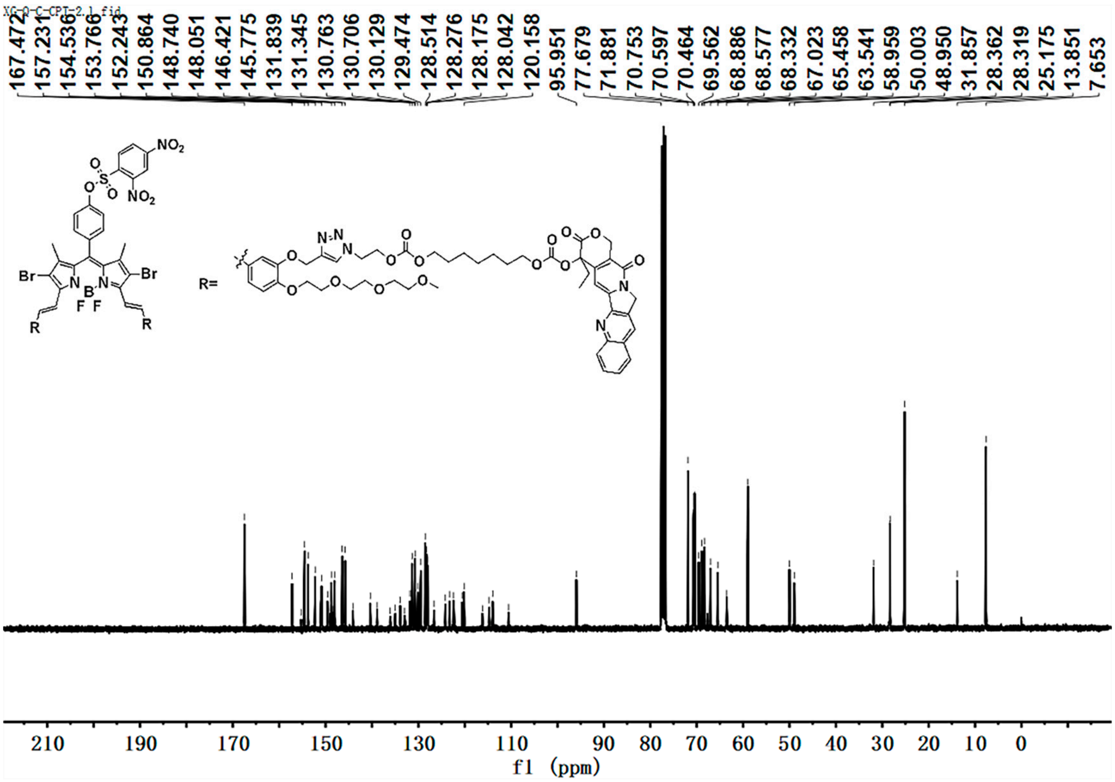

Figure S16. ^{13}C NMR spectrum of BCC in $CDCl_3$.

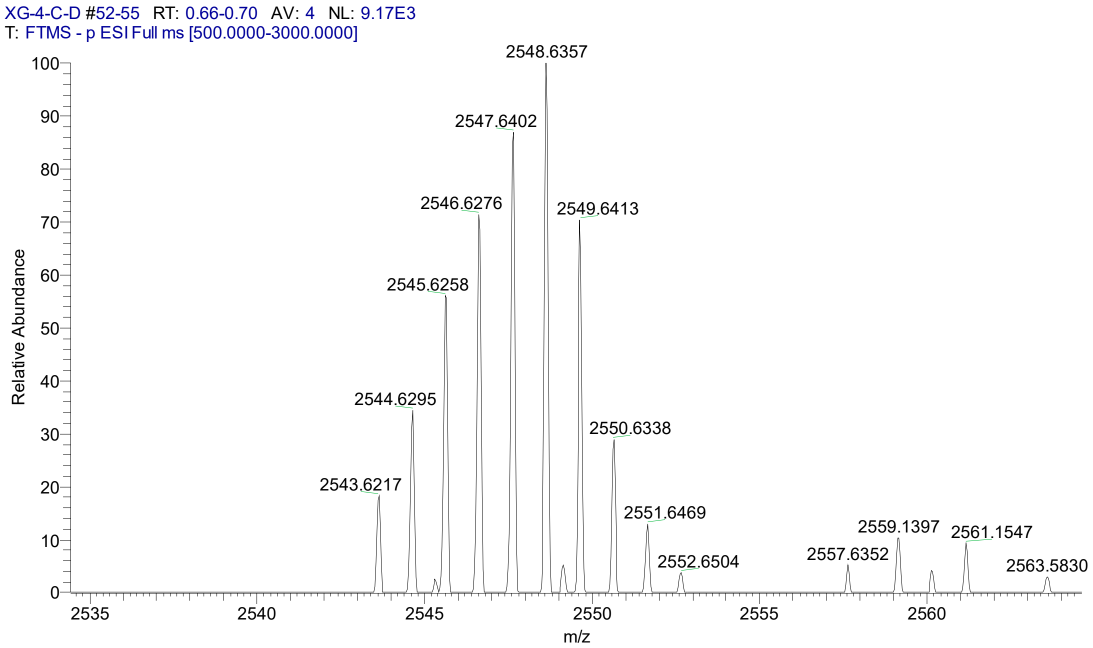

Figure S17. HRMS spectrum of BCC.
